# Transcatheter Edge-to-Edge Repair After Patent Foramen Ovale and Atrial Septal Defect Closure

**DOI:** 10.1016/j.jaccas.2026.107476

**Published:** 2026-04-24

**Authors:** Takaaki Sato, Takao Morikawa, Tomofumi Nakatsukasa, Teiji Akagi, Arudo Hiraoka, Misako Toki, Atsushi Hirohata

**Affiliations:** a Department of Cardiovascular Medicine, The Sakakibara Heart Institute of Okayama, Okayama, Japan; b Department of Cardiovascular Surgery, The Sakakibara Heart Institute of Okayama, Okayama, Japan; c Department of Clinical Laboratory, Sakakibara Heart Institute of Okayama, Okayama, Japan

**Keywords:** mitral regurgitation, septal closure device, transcatheter edge-to-edge repair, VersaCross

## Abstract

**Background:**

Transseptal puncture is essential for transcatheter edge-to-edge repair (TEER) but is challenging in patients with previously implanted septal closure devices.

**Case Summary:**

A 79-year-old woman with prior transcatheter closure of both a patent foramen ovale and an atrial septal defect developed severe degenerative mitral regurgitation (MR). Transseptal access was considered difficult due to the 2 closure devices. However, chronic MR caused enlargement of the left atrium and interatrial septum, creating a narrow but feasible puncture zone. Using the VersaCross system, transseptal puncture was achieved, allowing TEER completion.

**Discussion:**

Left atrial and septal enlargement from MR can create puncturable spaces despite prior septal closure devices. The VersaCross system enables controlled septal crossing, expanding the feasibility of TEER in anatomically complex patients.

**Take-Home Message:**

Chronic atrial remodeling secondary to severe MR may facilitate transseptal access after septal closure, and the VersaCross system can support safe puncture in challenging TEER cases.

## Visual Summary

**Figure:**
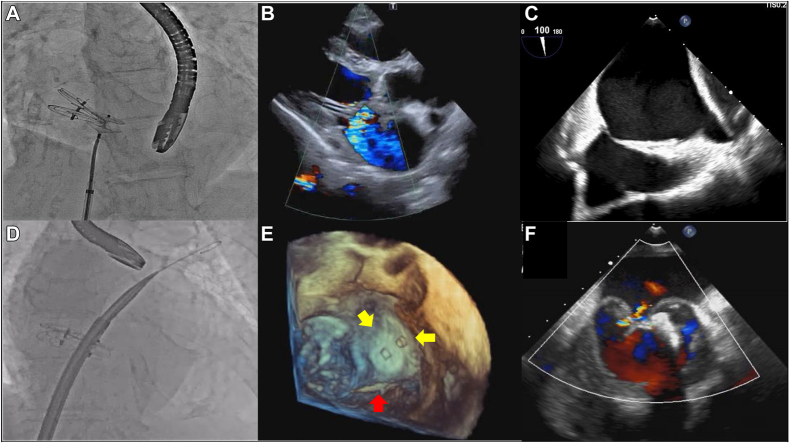

## History of Presentation

**Figure 1:**
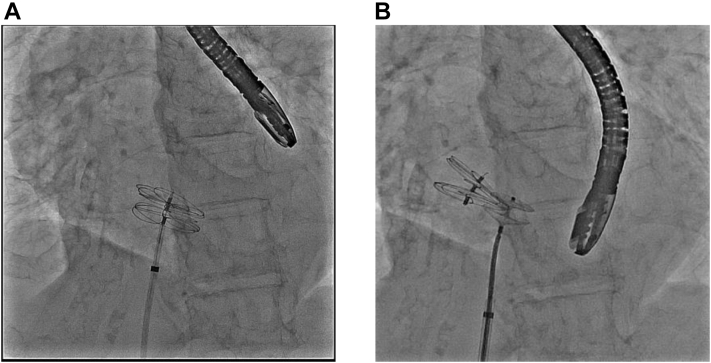
Images After Implantation of Septal Closure Devices (A) Fluoroscopic image showing patent foramen ovale closure using a Gore Cardioform occluder. (B) Fluoroscopic image demonstrating additional atrial septal defect closure with an Amplatzer Cribriform device using the sandwich technique.

A 79-year-old woman presented with persistent dyspnea and hypoxemia in the upright position, which improved when recumbent. Further evaluation revealed a patent foramen ovale (PFO) and an atrial septal defect (ASD) with right-to-left shunting. She was diagnosed with platypnea-orthodeoxia syndrome secondary to the coexistence of PFO and ASD. To relieve her symptoms, transcatheter closure of both the PFO and ASD was performed. As both defects could not be closed with a single device, 2 occluders were deployed: a 30-mm Cardioform device (Gore Medical) for the PFO and an 18-mm Amplatzer Cribriform device (Abbott Cardiovascular) for the ASD, using the sandwich technique (Figures 1A and 1B). At that time, no significant mitral regurgitation (MR) requiring treatment was observed.

One year later, she presented with a 1-month history of exertional dyspnea and bilateral leg edema. Her symptoms had gradually worsened, and she was referred to our hospital. Transthoracic echocardiography (TTE) revealed severe MR due to P1 leaflet prolapse with ruptured chordae tendineae, and she was admitted for treatment. There was no history of abrupt symptom onset suggestive of acute MR. No evidence of ischemic heart disease, infective endocarditis, or recent chest trauma was identified, and the underlying cause of the chordal rupture could not be definitively determined.

## Past Medical History

The patient had a 10-year history of hypertension, which was well controlled with azilsartan. She had no history of other surgical procedures apart from the septal closure.

## Differential Diagnosis

Differential diagnoses included MR due to chordal rupture, rheumatic disease, and mitral annular calcification.

## Investigations

**Figure 2:**
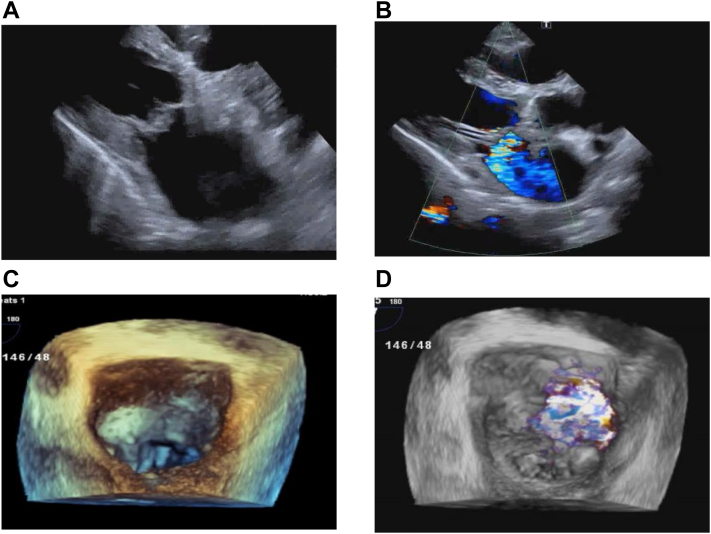
Echocardiographic Assessment of Mitral Regurgitation (A) TTE image in the parasternal long-axis view demonstrating P1 leaflet prolapse. (B) Color Doppler TTE in the parasternal long-axis view showing severe MR. (C) Three-dimensional TEE en face view of the mitral valve illustrating the P1 prolapse. (D) Three-dimensional TEE in the commissural view showing severe MR. MR = mitral regurgitation; TEE = transesophageal echocardiography; TTE = transthoracic echocardiography.

TTE revealed a large P1 leaflet prolapse, resulting in predominantly medially directed severe MR from the same segment. Transesophageal echocardiography demonstrated severe MR due to P1 leaflet prolapse with chordal rupture (Figures 2A to 2D). The MR was eccentric, making quantification of the effective regurgitant orifice area challenging; the mitral valve area measured 5.6 cm^2^. No shunt flow was observed across the interatrial septum.

Although no standardized method exists for quantifying interatrial septal dimensions, septal length was assessed using TTE in the apical four-chamber view. The distance from the mitral annular attachment to the atrial roof along the interatrial septum was measured to allow reproducible comparison. Using this method, the interatrial septal dimension increased from 51.2 mm before the development of MR to 59.5 mm at the time of hospitalization for severe MR. Left atrial size was also assessed by TTE in the apical four-chamber view. Prior to the development of MR, the left atrial dimensions measured 41 × 57 mm with a left atrial volume index of 37.0 mL/m^2^. At the time of hospitalization for severe MR, the left atrium was markedly enlarged, measuring 54 × 75 mm with a left atrial volume index of 102.7 mL/m^2^.

**Figure 3:**
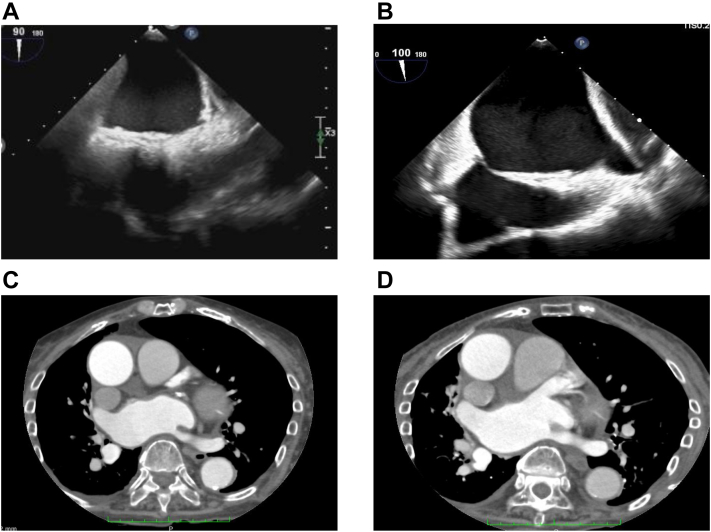
Comparison of Interatrial Septum and Left Atrial Enlargement Between the Prior and Current Procedures (A) TEE in the bicaval view immediately after septal device implantation, showing the interatrial septum almost entirely occupied by the closure devices. (B) TEE in the bicaval view at the time of transcatheter edge-to-edge repair, demonstrating stretching of the interatrial septum. (C) CT imaging obtained at the time of septal closure, showing normal left atrial dimensions. (D) CT imaging 1 year later, demonstrating marked left atrial enlargement and stretching of the interatrial septum. CT = computed tomography; TEE = transesophageal echocardiography.

Transseptal puncture, an essential step for transcatheter edge-to-edge repair (TEER), was anticipated to be challenging due to the presence of 2 previously implanted devices. However, compared with the prior procedure, left atrial enlargement and interatrial septal stretching associated with severe MR were evident on TTE. These findings were further confirmed by transesophageal echocardiography and computed tomography, allowing identification of a feasible puncture site just beneath the closure devices (Figures 3A to 3D).

Considering the high surgical risk for mitral valve replacement (Society of Thoracic Surgeons predicted mortality 7.65%), and after confirming that TEER was technically feasible, we decided to proceed with TEER after a heart team discussion.

## Management

The procedure was performed under general anesthesia with continuous transesophageal echocardiographic guidance. Right femoral venous access was obtained, and preclosure was performed using a Perclose ProStyle device (Abbott Cardiovascular). A 16-F sheath was then inserted, and transseptal puncture was subsequently performed.

**Figure 4:**
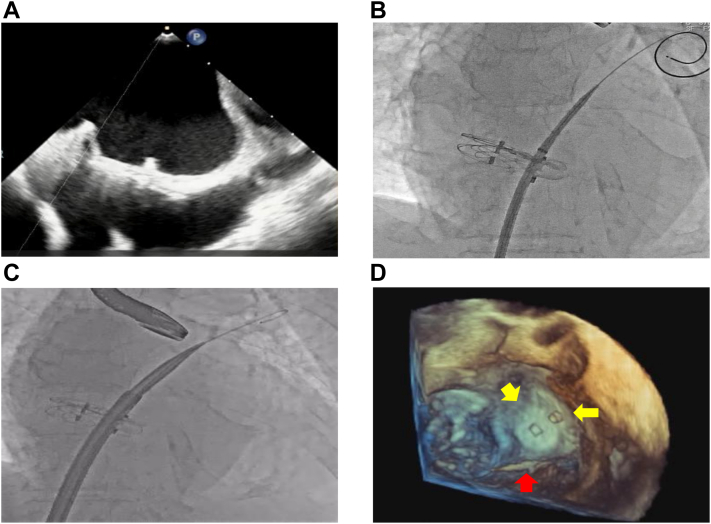
Images Demonstrating Transseptal Puncture and Device Passage (A) TEE bicaval view showing effective tenting of the inferoposterior interatrial septum with the SL-0 sheath. (B) Fluoroscopic image demonstrating successful passage of the SL-0 sheath across the interatrial septum following puncture. (C) Fluoroscopic image showing passage of the PASCAL Ace delivery system through the interatrial septum. (D) Three-dimensional TEE image showing the PASCAL Ace guiding sheath (red arrow) crossing the interatrial septum, with 2 previously implanted septal closure devices also visualized (yellow arrows). TEE = transesophageal echocardiography.

**Figure 5:**
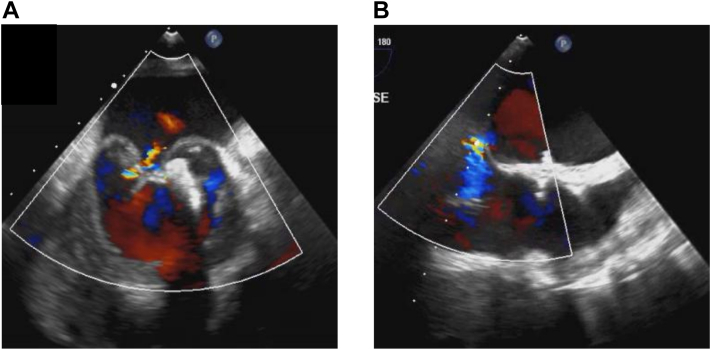
Echocardiographic Findings After Transcatheter Edge-to-Edge Repair (A) TEE image showing mild residual mitral regurgitation after transcatheter edge-to-edge repair. (B) TEE image confirming the absence of residual iatrogenic atrial septal defect following removal of the guide catheter. TEE = transesophageal echocardiography.

Because only a narrow inferoposterior septal space remained available beneath the 2 closure devices, the conventional approach using an radiofrequency transseptal needle (Baylis) was technically challenging. Therefore, atrial septal puncture was performed using the VersaCross system (Boston Scientific) instead of the radiofrequency transseptal needle. By advancing VersaCross first, the SL-0 sheath was guided from the inferior vena cava toward the fossa ovalis, enabling successful transseptal puncture at the inferoposterior portion of the interatrial septum without interference (Figures 4A to 4D, Video 1, Video 2, Video 3). Subsequently, we performed TEER in the standard manner, and a PASCAL Ace (Edwards Lifesciences) device was deployed at the site of the P1 leaflet prolapse. The prolapse was adequately controlled, resulting in a reduction of MR to mild, with a mean mitral valve pressure gradient of 0.8 mm Hg (Figure 5A). Left atrial pressure also decreased, allowing safe release of the device. The procedure was completed without residual iatrogenic ASD following removal of the guide catheter (Figure 5B). The right femoral vein was closed, and the procedure was concluded.

## Outcome and Follow-Up

The patient experienced marked improvement in dyspnea after the procedure. TTE performed on postoperative day 1 showed mild MR with a mean mitral valve pressure gradient of 0.7 mm Hg. The patient had an uneventful postoperative course and was discharged on postoperative day 4. At the 1-month follow-up, TTE demonstrated moderate MR, representing a mild increase compared with the immediate postprocedural findings. Despite this, the patient remained clinically stable without recurrence of heart failure symptoms.

## Discussion

TEER has been reported to be a safe and effective treatment for patients with severe primary or secondary MR who are at high surgical risk,^1^, ^2^, ^3^, ^4^ and it has become an increasingly accepted therapeutic option in this population. Transseptal puncture is an essential step in performing TEER; however, it becomes particularly challenging in patients with previously implanted septal closure devices. In the present case, 2 closure devices occupied a large portion of the interatrial septum, initially suggesting that transseptal puncture would be technically infeasible. However, successful septal crossing and TEER were achieved owing to anatomical remodeling induced by chronic severe MR.

It has been well established that MR imposes both volume and pressure overload on the left atrium, leading to progressive dilation and remodeling of the left atrium and, to some extent, the interatrial septum.^5^ This remodeling process represents an adaptive yet pathological response to long-standing hemodynamic stress. In this case, left atrial enlargement and septal stretching created a narrow but distinct puncturable zone at the inferoposterior septum beneath the previously implanted closure devices, which had not been accessible during the initial septal closure procedure. We therefore consider the enlargement of the interatrial septum to be a key anatomic change that rendered transseptal puncture technically possible in this otherwise prohibitive situation. Thus, chronic MR-induced remodeling paradoxically enhanced the feasibility of TEER in this patient.

From a technical standpoint, several procedural strategies were implemented to take advantage of this limited opportunity. First, the use of the VersaCross system was pivotal. Unlike the conventional approach, which requires advancing the SL-0 sheath into the superior vena cava and manipulating it downward with an radiofrequency transseptal needle, the VersaCross system allowed direct and stable advancement of the SL-0 sheath from the inferior vena cava toward the inferoposterior interatrial septum. This direct trajectory minimized interference from the closure devices and enabled precise puncture at the limited available site. Prior clinical studies have demonstrated that radiofrequency wire–based transseptal systems, such as VersaCross, can streamline transseptal workflows and enable faster access to the left atrium compared with conventional mechanical needle techniques, potentially improving procedural efficiency.^6^

Second, the PASCAL system was chosen instead of the MitraClip system (Abbott Cardiovascular). The PASCAL steerable guide catheter has a smaller outer diameter (22-F vs 24-F for the MitraClip G4), which reduced the spatial requirement for transseptal access and minimized the risk of device interference during catheter navigation and delivery system manipulation. This design advantage was particularly beneficial given the restricted puncture angle and narrow septal window in this case.

Several cases have been reported in which transseptal puncture was successfully performed in the presence of either an ASD or a PFO closure device.^7^, ^8^, ^9^ In addition, a prior report described successful transseptal puncture in a patient with multiple septal occluder devices.^10^ In contrast, the present case is unique in that it highlights chronic MR-related left atrial remodeling. This remodeling was accompanied by stretching of the interatrial septum, which served as a key anatomic factor enabling transseptal access for TEER despite the presence of both ASD and PFO closure devices.

This case represents a rare instance in which left atrial remodeling secondary to severe MR enabled successful transseptal puncture and completion of TEER despite the presence of septal closure devices, providing important insights for reconsidering treatment strategies in anatomically complex settings.

## Conclusions

This case demonstrates that chronic left atrial remodeling secondary to severe MR may, paradoxically, create an opportunity for transseptal puncture despite the presence of septal closure devices. Furthermore, the use of the VersaCross system may enhance procedural feasibility in anatomically complex TEER cases.

**Visual Summary:**
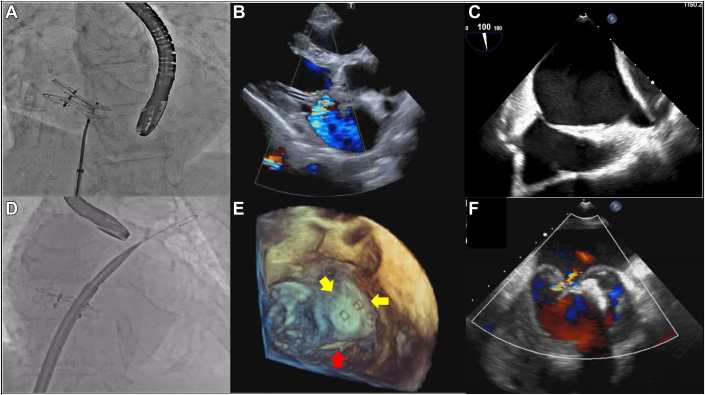
Key Imaging Findings in TEER After Prior Septal Closure (A) Fluoroscopic image from 1 year earlier showing transcatheter closure of the patent foramen ovale and atrial septal defect using 2 occluder devices. (B) Transthoracic echocardiography on day 1 demonstrating severe degenerative MR due to P1 prolapse with ruptured chordae. (C) TEE in the bicaval view at the time of TEER, demonstrating stretching of the interatrial septum. (D) Fluoroscopic image showing passage of the PASCAL Ace delivery system through the interatrial septum. (E) Three-dimensional TEE image showing the PASCAL Ace guiding sheath (red arrow) crossing the interatrial septum, with 2 previously implanted septal closure devices also visualized (yellow arrows). (F) TEE image showing mild residual mitral regurgitation after transcatheter edge-to-edge repair. MR = mitral regurgitation; TEE = transesophageal echocardiography; TEER = transcatheter edge-to-edge repair.

## Funding Support and Author Disclosures

The authors have reported that they have no relationships relevant to the contents of this paper to disclose.

**Take-Home Messages**

• Chronic left atrial remodeling secondary to severe mitral regurgitation may create new puncturable spaces within the interatrial septum.
• In anatomically challenging transcatheter edge-to-edge repair cases, the VersaCross system can facilitate safe and successful transseptal puncture.

## Appendix

Video 1 Advancement of the VersaCross System Toward the Interatrial Septum Fluoroscopic imaging demonstrating advancement of the VersaCross system from the inferior vena cava toward the interatrial septum.

Video 2 Transseptal Puncture Using the VersaCross System Fluoroscopic imaging showing transseptal puncture at the inferoposterior aspect of the interatrial septum and advancement of the VersaCross system into the left atrium.

Video 3 Passage of the PASCAL Guiding Sheath Into the Left Atrium Fluoroscopic imaging demonstrating successful passage of the PASCAL guiding sheath into the left atrium following transseptal puncture.
